# The Principles and Practice of Bandaging

**Published:** 1902-09

**Authors:** 


THE PRINCIPLES AND PRACTICE OF BANDAGING. By Gilwym G. Davis, M. D., University of Pennsylvania and Gottingen; Member of the Royal College of Surgeons, England; Assistant Professor of Applied Anatomy, University of Pennsylvania; Surgeon to the Episcopal, St. Joseph's, and Orthopedic Hospitals. With 163 illustrations from original drawings by the author, and 146 pages of text. J. Blackiston's Son & Co., Philadelphia, 1902. Cloth, $1.50 net.

This little volume represents a distinct improvement, both in the matter of texts and illustrations, over any other work of the kind with which we are familiar. The language is simple and direct, and the book's freedom from technical terms and involved sentences will make it of especial value to beginners and to those who require such a work for quick reference.

The art of bandaging has received far too little attention during recent years. It now seems to be the practice with many surgeons to wind a bandage aimlessly, without any idea of proper sequence in making the turns, the accommodating gauze bandage being trusted to conceal the operator's ignorance. Dr. Davis's little book is just the aid that such men need. R.

